# 

**Published:** 1861-08

**Authors:** 


### CONTENTS:

### ORIGINAL COMMUNICATIONS.

PAGE.

ART. I.—Case of Ovarian Dropsy successfully treated by Iodine Injections. By W. C. Smith, of Grantville, Ga. . . . . . . . . . . . . . . . . . 690

ART. II.—Scirrhous Tumor of the Lip. By. G. W. Powell, M. D., of Childersburg, Alabama . . . . . . . . . . . . . . . . . . . . . . . . . . . . . . . . . 695

ART. III.—Curious Symptoms from Worms. By H. L. Wilson, M.D., of Atlanta, Ga . . . . . . . . . . . . . . . . . . . . . . . . . . . . . . . . . . . . . . . . 703

### SELECTIONS.

Observations on the Treatment of Asthma. By T. L. Pridham, of Bidford, North Devon . . . . . . . . . . . . . . . . . . . . . . . . . . . . . . . . . . 705

Turning by External Manipulation . . . . . . . . . . . . . . . . . . . . . . . . . . 719

Uterine Lucorrhæ of Old Women . . . . . . . . . . . . . . . . . . . . . . . . . . . 722

Letter from S. S. Satchwell, M. D . . . . . . . . . . . . . . . . . . . . . . . . . . . 725

On the Effects of Alcohol, or Spirit-Drinking, in Diseases of the Liver. Delivered at Westminster Hospital, by W. R. Basham, M.D., Physician to the Hospital . . . . . . . . . . . . . . . . . . . . . . . . . . . . . 731

Whisky, as a Prophylatic in Typhoid Fever. By Hugh Kelly, M.D., of Iredell County, N. C . . . . . . . . . . . . . . . . . . . . . . . . . . . . . . . . . . . . 743

### EDITORIAL AND MISCELLANEOUS.

Atlanta Medical College—Our Journal, etc . . . . . . . . . . . . . . . . . . . . . . 746

Shelby Medical College, Nashville, Tenn . . . . . . . . . . . . . . . . . . . . . . . 750

